# Controlled meal frequency without caloric restriction alters peripheral blood mononuclear cell cytokine production

**DOI:** 10.1186/1476-9255-8-6

**Published:** 2011-03-07

**Authors:** Vishwa Deep Dixit, Hyunwon Yang, Khaleel S Sayeed, Kim S Stote, William V Rumpler, David J Baer, Dan L Longo, Mark P Mattson, Dennis D Taub

**Affiliations:** 1 Laboratory of Molecular Biology and Immunology, National Institute on Aging, National Institutes of Health, (251 Bayview Boulevard), Baltimore, MD, (21224), USA; 2 Laboratory of Neurosciences, National Institute on Aging, National Institutes of Health, (251 Bayview Boulevard), Baltimore, MD, (21224), USA; 3 Beltsville Human Nutrition Research Center, United States Department of Agriculture, Agriculture Research Service, (10300 Baltimore Avenue, Beltsville, Maryland, (20705), USA; 4 Laboratory of Neuroendocrine-Immunology, Pennington Biomedical Research Center, Baton Rouge, LA 70808, USA

## Abstract

**Background:**

Intermittent fasting (IF) improves healthy lifespan in animals by a mechanism involving reduced oxidative damage and increased resistance to stress. However, no studies have evaluated the impact of controlled meal frequency on immune responses in human subjects.

**Objective:**

A study was conducted to establish the effects of controlled diets with different meal frequencies, but similar daily energy intakes, on cytokine production in healthy male and female subjects.

**Design:**

In a crossover study design with an intervening washout period, healthy normal weight middle-age male and female subjects (n = 15) were maintained for 2 months on controlled on-site one meal per day (OMD) or three meals per day (TMD) isocaloric diets. Serum samples and peripheral blood mononuclear cells (PBMCs) culture supernatants from subjects were analyzed for the presence of inflammatory markers using a multiplex assay.

**Results:**

There were no significant differences in the inflammatory markers in the serum of subjects on the OMD or TMD diets. There was an increase in the capacity of PBMCs to produce cytokines in subjects during the first month on the OMD or TMD diets.

Lower levels of TNF-α, IL-17, MCP-1 and MIP-1β were produced by PBMCs from subjects on the OMD versus TMD diet.

**Conclusions:**

PBMCs of subjects on controlled diets exhibit hypersensitivities to cellular stimulation suggesting that stress associated with altered eating behavior might affect cytokine production by immune cells upon stimulation. Moreover, stimulated PBMCs derived from healthy individuals on a reduced meal frequency diet respond with a reduced capability to produce cytokines.

## Introduction

It has been hypothesized that due to limited availability of food throughout the majority of human evolution, the body was more adapted towards intermittent feeding rather than to regular meal intervals as currently practiced in the developed world [1]. Regular access to high calorie diets has contributed to an increase in obesity and associated increases in morbidity and mortality [2]. Studies of obesity and its antithesis, caloric restriction (CR), in humans and animals have provided insight into the cellular and molecular mechanisms underlying normal aging and chronic diseases including type 2 diabetes, cardiovascular disease, cancers and neurodegenerative disorders [3-5]. The multi-system pleiotropic effects of dietary restriction also extend to the immune system. Many studies suggest that long-term CR improves several components of immune function including responses of T cells to mitogens, natural kill cell (NK) activity, cytotoxic T lymphocyte (CTL) activity and the ability of mononuclear cells to produce pro-inflammatory cytokines [6-8]. CR attenuated the age-associated increase in ratio of memory to naïve T cells in monkeys, and this was associated with a reduction in the pro-inflammatory cytokines, TNF-α and IL-6 [9]. It has been suggested that a prominent immune-enhancing effect of CR on NK cells and CTL mediates, in part, the reduced incidence of tumors in mice maintained on CR diets [10,11].

Data from controlled studies in rodents suggest that intermittent fasting (IF) can protect against age-related diseases and can extend lifespan, and that at least some of the beneficial effects of IF may be independent of calorie intake [1,4]. For example, alternate day fasting protected neurons in the brains of mice against dysfunction and degeneration in models of Parkinson's and Alzheimer's diseases and stroke [12-14]. IF resulted in improved glucose regulation and cardiovascular function [15,16] and protected the heart against ischemia reperfusion injury [17]. The latter study provided evidence that the cardioprotective effect of IF is associated with an attenuation of tissue inflammation. Increasing evidence suggests that the signaling mechanisms that regulate energy metabolism and immune function are tightly coupled to each other [18,19]. For example, fasting can significantly attenuate inflammation and the development of autoimmune encephalomyelitis [20]. In addition, the orexigenic hormone ghrelin can act on various immune cell subsets and inhibit pro-inflammatory cytokine production [21]. Furthermore, genomic profiling studies in rodents revealed that CR can reverse the increased inflammation associated with aging [22] and inhibit the release of proinflammatory mediators from macrophages [23].

Recent findings from the Comprehensive Assessment of Long-Term Effects of Reducing Intake of Energy (CALERIE) study suggest that CR has effects on energy metabolism and disease risk in humans that are similar to those seen in rodents [24]. In humans, long-term CR was reported to be highly effective in reducing the risk for atherosclerosis and associated pro-inflammatory markers [25], and moderate CR improved cell-mediated immunity [26]. In contrast to the increasing literature describing effects of CR on the immune system, there have been no reports of studies of how reduced meal frequency/IF affects immune function. It was recently reported that an alternate day calorie restriction IF dietary regimen resulted in a marked improvement in the symptoms of asthma patients, and an associated reduction in serum markers of oxidative stress and inflammation [27]. However, the IF diet included a large reduction in calorie intake such that the relative contributions of CR and fasting to the outcomes is unknown.

We have previously reported on a human meal frequency study in which the daily calories were held constant between two diets that differed only in meal frequency (3 smaller meals versus one large meal). In this study, a large number of physiological variables were measured, including heart rate, body temperature and blood chemicals and many of these were unaffected by altering meal frequency [28]. However, when on 1 meal per day, subjects did exhibit a significant reduction of fat mass and significant increases in levels of total, low-density lipoprotein, and high density lipoprotein cholesterol. Moreover, in this same study, Carlson and coworkers [29] demonstrated that the morning glucose tolerance was found to be impaired in subjects consuming 1 meal per day compared with 3 meals per day. Fasting (morning) plasma glucose levels were also significantly elevated in subjects when they were consuming 1 meal per day (OMD) compared with 3 meals per day (TMD). This OMD diet effect on glucose tolerance was rapidly reversed upon return to the TMD diet, indicating that the diet had no long-lasting effect on glucose metabolism. Interestingly, there were no significant effects of meal frequency on plasma levels of ghrelin, adiponectin, resistin or BDNF.

In a follow-up to these studies, we have here examined the impact of different meal frequencies (without a difference in calorie intake) on plasma inflammatory markers (CRP, sgp130, visfatin) and activation-induced PBMC cytokine expression in normal weight human male and female subjects. Our data suggest that a change in diet causes a transient increase in TCR- and TLR4-mediated pro-inflammatory cytokine production by peripheral blood mononuclear cells (PBMCs), and that the magnitude of these alterations is less when subjects consume OMD vs. TMD.

## Subjects and Methods

### Subjects, Study Design and Diets

Details of the subject population, selection criteria and study design have been reported [28]. Briefly, subjects were healthy 40-50 year-old males and females with a body mass index (BMI) between 18 and 25 kg/m^2 ^with a usual eating pattern of TMD. The experimental protocol was approved by the Johns Hopkins University Committee on Human Research and the MedStar Research Institute Institutional Review Board, and all subjects gave their informed consent. As this is the first study of its kind, there is no historical data for comparison in design and to determine wash out periods. This study was designed based on animal studies in which we found that many physiological variables (heart rate, blood pressure, insulin levels) returned to baseline levels within 2-4 weeks of wash-out [1,12-14]. Thus, the subjects in this study were divided into two controlled diet groups, a TMD diet and an OMD diet, in a washout and crossover design - 2 months on diets, 2 months off diets, crossover 2 months on diet - with the study lasting 6 months. During both 2-month controlled diet periods, each subject consumed dinner at the Human Study Facility under the supervision of a registered dietitian. Only foods provided by the Human Study Facility were allowed to be consumed during the study. Subjects were allowed unlimited amounts of caloric-free liquids and foods. Prior to initiation of the experimental diets, the energy requirements for weight maintenance were calculated for each subject using the Harris- Benedict formula, which estimates basal energy expenditure, and multiplied by an activity factor of 1.3-1.5. This formula has proven successful in estimating weight-maintenance energy requirements at our facility. For the entire study the average daily calorie intakes were 2364 kcal in the 1 meal/d diet and 2429 kcal in the 3 meals/d diet. More details on the diet composition and methods used to evaluate compliance with the diets are reported elsewhere [28,29].

As for the population of subjects examined in this study, the number of subjects (n) examined for each stage of the study are as follows: Pre-treatment/Baseline (n = 15), 1 meal/1 month (n = 8), 1 meal/2 month (n = 12), Off-diet (n = 12), 3 meals/1 month (n = 12) and 3 meal/2 month (n = 12). Complete data were analyzed and are presented for 15 subjects. In the TMD diet arm, 1 subject withdrew because of food dislikes. During the OMD, 5 subjects withdrew because of scheduling conflicts and health problems unrelated to the study. Only 1 of the 5 subjects withdrew specifically because of an unwillingness to consume the 1 meal/d diet.

### Separation and stimulation of peripheral blood mononuclear cells (PBMCs)

The PBMCs were separated from fresh heparinized blood of healthy adult donors using Ficoll density gradient centrifugation, followed by extensive washing in phosphate-buffered saline (PBS). The erythrocytes were removed by hypotonic shock (ACK lysis buffer, Quality Biological, Bethesda, MD). The PBMCs were subsequently cultured in serum-free medium (AIM-V) and stimulated with either plate-bound anti-CD3 mAb (200 ng/ml) or *E. coli *LPS (10 μg/ml) for 24 hours as described previously [21].

### Cytokine analysis

Serum and cell culture supernatants were analyzed for cytokines using Bio-Plex Cytokine 17-Plex Panel according to manufacturer's instructions (Biorad Laboratories, Hercules, CA).

### Real Time PCR analysis

The PBMCs were lysed in RNA lysis buffer and total RNA was extracted from control and stimulated cells using a QIAshredder kit (QIAgen). RNA (500 μg) and oligo-dT primers were used to synthesize single-stranded cDNA. PCR was then performed using SYBR green Master Mix (Applied Biosystems, Foster City, California, USA), 1 μl cDNA, and exon spanning gene-specific primers. Thermal cycling was performed using the Applied Biosystems GeneAmp 7700 Sequence Detector.

### Statistical Analysis

Data are presented as the mean and SEM. An analysis of variance appropriate for a 2 period crossover study with repeated measures within period was used to evaluate meal frequency effects on outcome variables. The Student-Newman-Keuls test was employed to test the significance of difference observed in the two study groups.

## Results

### Serum Markers of Inflammation

**Figure 1:**
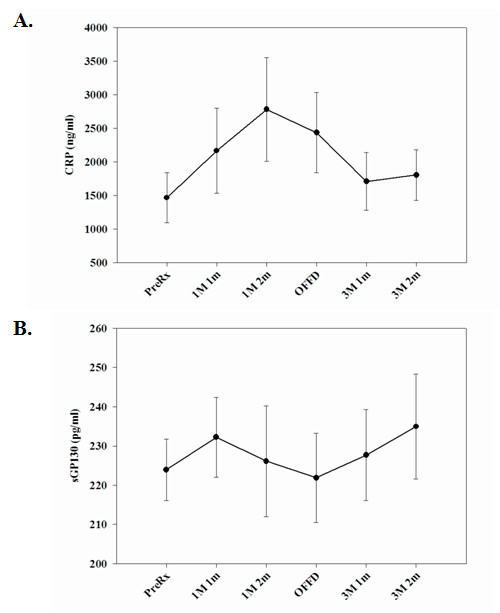
**Serum CRP and soluble gp130 levels from OMD and TMD fed subjects at various time points during the study**. **(A.) **Serum CRP and **(B.) **soluble gp130 (sGP130) concentrations were examined at the indicated time points. The data are expressed in pg/ml (+/- SEM). OMD, one meal per day controlled diet; TMD, three meals per day controlled diet. The numbers of subjects (n) examined for each stage of the study are as follows: Pre-treatment/Baseline (n = 15), 1 meal/1 month (n = 8), 1 meal/2 month (n = 12), Off-diet (n = 12), 3 meals/1 month (n = 12) and 3 meal/2 month (n = 12).

**Figure 2:**
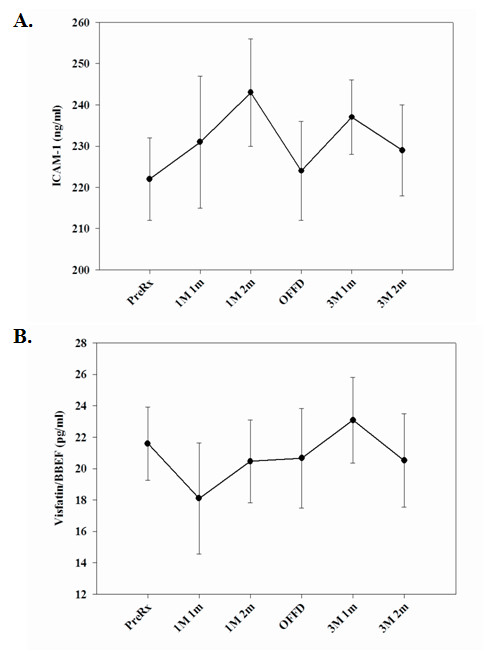
**Serum CRP and soluble gp130 levels from OMD and TMD fed subjects at various time points during the study**. ***(*****A.) **Serum intercellular adhesion molecule-1 (ICAM-1) and **(B.) **visfatin concentrations were examined at the indicated time points. The data are expressed in pg/ml (+/- SEM). The numbers of subjects (n) examined for each stage of the study are as follows: Pre-treatment/Baseline (n = 15), 1 meal/1 month (n = 8), 1 meal/2 month (n = 12), Off-diet (n = 12), 3 meals/1 month (n = 12) and 3 meal/2 month (n = 12).

Measurement of C-reactive protein (CRP), ICAM-1, VCAM-1 and soluble gp130 proteins in the peripheral circulation reflect the basal inflammatory state [30]. CRP levels were elevated in subjects when they were on the OMD diet compared to the TMD diet (Figure 1A). There were no significant effects of diet on serum levels of sgp130 (Figure 1B), ICAM-1 (Figure 2A) or VCAM-1 (data not shown). In addition, diet demonstrated no significant effects on levels of circulating visfatin (nicotinamide phosphoribosyltransferase; Pre-B cell colony enhancing factor) (Figure 2B), a recently identified adipocytokine that has insulin-mimetic effects [31] and pro-inflammatory properties [32,33].

### Cytokine Secretion from Peripheral Blood Mononuclear Cells

**Figure 3:**
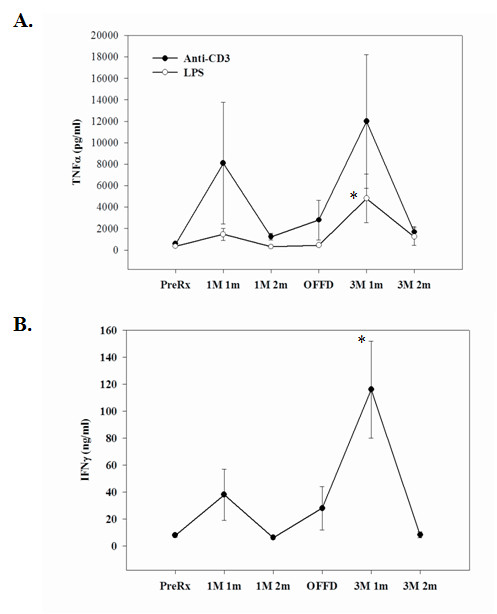
**Stimulated PBMCs derived from subjects on OMD and TMD diets were examined for TNF-α and IFN-γ expression**. Peripheral blood mononuclear cells derived from OMD and TMD diets were stimulated ex-vivo with anti-CD3 mAb or LPS. In both the study groups, there was a statistically significant (p < 0.05) increase in TNF-α and levels in culture supernatants at one month after initiation of dietary regimens. **(A) **LPS-induced TNF-α release at 1 month time point was significantly lower in OMD fed subjects compared to the TMD group. No significant differences could be detected at other time points and in response to anti-CD3 mAb stimulation. **(B) **T cell activation by TCR-dependent mechanisms (anti-CD3 mAb) results in a lower IFN-γ release at one month time point in subjects fed OMD versus those fed TMD. The data are expressed in pg/ml (+/- SEM). The numbers of subjects (n) examined for each stage of the study are as follows: Pre-treatment/Baseline (n = 15), 1 meal/1 month (n = 8), 1 meal/2 month (n = 12), Off-diet (n = 12), 3 meals/1 month (n = 12) and 3 meal/2 month (n = 12).

In the absence of antigenic challenge, immune cells produce negligible or low levels of pro-inflammatory cytokines. In an effort to understand the impact of meal frequency on lymphocyte responsiveness to an immune challenge, we isolated PBMC from subjects on OMD and TMD diets and challenged them ex-vivo. Due to the limitations on volume of blood collections from subjects and the availability of buffy coats in the study, isolation of specific immune cell subsets was not feasible. In an effort to understand the cytokine secretory responses of immune cell subsets, LPS was utilized to stimulate B cells and monocytes via toll-like receptor 4 (TLR4), while the T cells in the mixed PBMC populations were specifically activated by TCR ligation. TNF-α production induced by LPS and anti-CD3 mAb treatment was significantly greater during the first month on either the OMD or TMD controlled diet periods compared to the pretreatment and washout time points (Figure 3A). The increase in TNF-α levels at the one month time point was followed by a return towards baseline during the subsequent one month of the both the TMD and OMD diet periods. However, the magnitude of the elevation of TNF-α level at one month was greater when the subjects ate TMD compared to OMD (Figure 3A).

Similar to TNF-α, there was a transient increase in the amount of Th-1 cytokine, IFN-γ secreted in response to anti-CD3 mAb stimulation in PBMC from subjects at 1 month after initiation of either OMD or TMD diets (Figure 3B). The magnitude of enhancement of IFN-γ production was significantly greater in subjects on TMD compared to OMD. Both basal and anti-CD3 mAb-stimulated production of IL-6 were elevated at the 1 month on-diet time point compared to the pretreatment, off-diet, and 2 month on-diet time points (Table 1). Anti-CD3 mAb-stimulated production of IL-1β by PBMCs was also significantly greater at the 1 month on-diet time point in both the OMD and TMD groups compared to other time points (Table 1). Compared to the pretreatment time point, the level of IL-2 produced in response to stimulation with anti-CD3 mAb was elevated at all of the other time points during the 6 month study period (Table 1). Levels of GM-CSF and G-CSF produced by PBMCs in response to stimulation with either anti-CD3 mAb or LPS treatment were significantly greater at the 1 month on-diet time points for both the OMD and TMD diet groups, with the levels being greatest when subjects were consuming TMD (Table 1). Levels of IL-10 produced in response to stimulation with anti-CD3 mAb were greatest at the 1 month on-diet time points for both the OMD and TMD groups (Table 1).

**Table 1:** Anti-CD3 mAb- and LPS-induced cytokine expression by PBMCs derived from OMD and TMD fed subjects at various time periods during the trial.

Cytokines	Pretreatment	1 meal, 1 month	1 meal, 2 months	Off diet	3 meals, 1 month	3 meals, 2 months
**Subject Number**	15	8	12	12	12	12

**IL-6 (pg/ml)** **Unstimulated**	520.5 ± 420.3	1290.2 ± 52.9	275.8 ± 52.9	871.6 ± 420.8	2653.8 ± 912.5	459.2 ± 154.3

**IL-6 (pg/ml)** **Anti-CD3 mAb**	1089.2 ± 195.6	32794.2 ± 17902.1	4163.2 ± 647.5	19669.1 ± 13184.2	61393 ± 34081	4291.8 ± 736.4

**IL-1β (pg/ml)** **Anti-CD3 mAb**	229.4 ± 47.2	3778.6 ± 2181.4	243.4 ± 61.5	1429.3 ± 897.6	4234.6 ± 1526.9	245.3 ± 59.8

**IL-2 (pg/ml)** **Anti-CD3 mAb**	28.3 ± 5.2	121.5 ± 78.3	85.4 ± 33.2	174.6 ± 146.3	146.5 ± 63.8	288.4 ± 139.6

**GM-CSF (pg/ml)** **Anti-CD3 mAb**	45.2 ± 4.2	279.8 ± 12.7	44.6 ± 7.8	347.3 ± 263.5	679.1 ± 259.6	109.3 ± 55.96

**GM-CSF (pg/ml)** **LPS**	63.2 ± 11.5	169.3 ± 96.8	74.6 ± 12.3	82.6 ± 22.3	934.6 ± 50.7	274.3 ± 169.5

**G-CSF (pg/ml)** **Anti-CD3 mAb**	98.4 ± 62.3	264.5 ± 146.3	55.6 ± 12.1	103.6 ± 62.2	405.9 ± 125.4	54.2 ± 16.2

**G-CSF (pg/ml)** **LPS**	1075.4 ± 221.1	7912 ± 5880.1	2195.3 ± 374.6	2669.4 ± 1008.6	10608.2 ± 4633.8	4466.1 ± 2213.5

**IL-10 (pg/ml)** **Anti-CD3 mAb**	270.5 ± 63.1	3960.1 ± 3103	381.7 ± 81.6	2055.4 ± 1699.5	4404.5 ± 1398.9	524.5 ± 212.6

^a ^Mean (pg/ml ± SEM) concentration of various cytokines measured in culture supernatants in response to LPS- and TCR-mediated activation stimuli.

**Figure 4:**
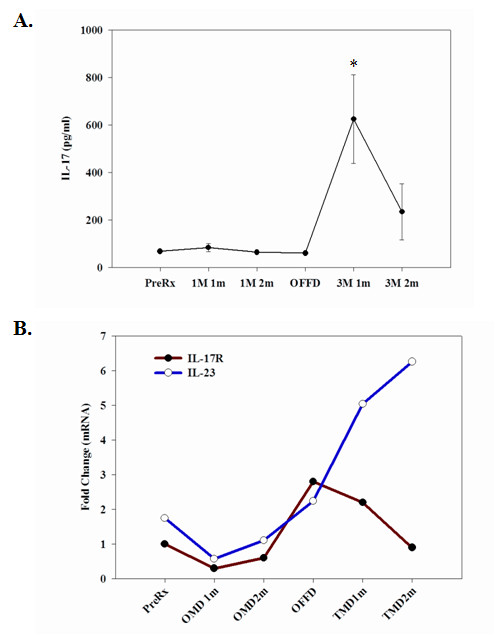
**Stimulated PBMCs derived from subjects on OMD and TMD diets were examined for IL-17 production and for mRNA expression of IL-17R and IL-23**. PBMCs isolated from subjects at the indicated time points were stimulated with anti-CD3 mAb antibody. **(A.) **The IL-17 secretion at 1 month time point was significantly lower in OMD fed subjects compared to TMD (p < 0.05). The data are expressed in pg/ml (+/- SEM). **(B.) **Equal amounts of cDNA from individual donors were analyzed for IL-17R and IL-23 mRNA levels using real-time RT-PCR. Each sample was run in duplicate and the threshold value (Ct) was normalized to GAPDH and is expressed as average fold change. The numbers of subjects (n) examined for each stage of the study are as follows: Pre-treatment/Baseline (n = 15), 1 meal/1 month (n = 8), 1 meal/2 month (n = 12), Off-diet (n = 12), 3 meals/1 month (n = 12) and 3 meal/2 month (n = 12).

A subset of IL-17 producing T (Th17) cells distinct from Th-1 or Th-2 cells has been described and shown to play a critical role in the induction of autoimmune diseases [34,35]. Interestingly, we observed a significantly higher production of IL-17 from anti-CD3 stimulated T cells in the subjects when on the TMD diet compared to the OMD diet (Figure 4A). The mRNA expression of IL-17 receptor from pooled cDNA samples of subjects in OMD and TMD did not show any significant changes. IL-23 has recently been reported to play a role in the development of IL-17-producing T helper cells [36]. In an effort to understand the possible mechanism responsible for increased IL-17 release in subjects on the TMD, we measured IL-23 mRNA levels by real-time PCR analysis in the anti-CD3 mAb activated PBMCs. We observed a 4- to 5-fold higher IL-23 mRNA expression in subjects when they were on the TMD diet compared to OMD diet (Figure 4B); consistent with the possibility that IL-23 regulates IL-17 expression.

**Figure 5:**
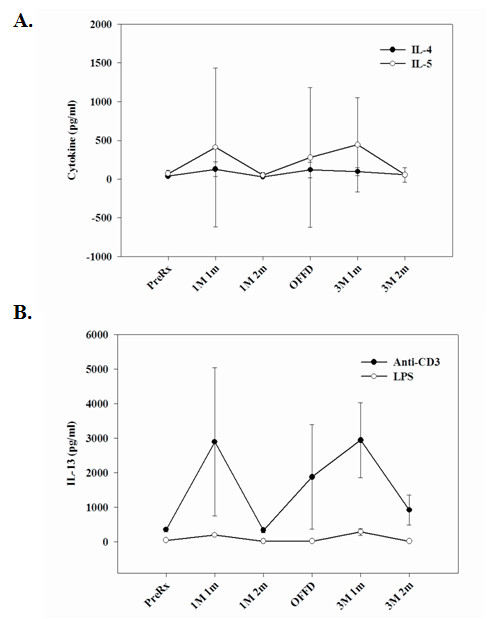
**Stimulated PBMCs derived from subjects on OMD and TMD diets were examined for IL-4, IL-5 and IL-13 expression**. **(A.) **The production of classical T helper-2 cytokines, IL-4 and IL-5 from anti-CD3 mAb stimulated PBMCs demonstrated no significant difference between OMD and TMD diet groups. **(B.) **There were no significant effects of diet on the capacity of PBMCs to release IL-13. The data are expressed in pg/ml (+/- SEM). The numbers of subjects (n) examined for each stage of the study are as follows: Pre-treatment/Baseline (n = 15), 1 meal/1 month (n = 8), 1 meal/2 month (n = 12), Off-diet (n = 12), 3 meals/1 month (n = 12) and 3 meal/2 month (n = 12).

The effects of diet on Th-1 and Th17 cytokine expression were not associated with any significant effect on the Th-2 cytokines IL-4 and IL-5 (Figure 5A) or IL-10 (Table 1). There were no statistically significant effects of diet on ant-CD3 mAb- or LPS-induced production of IL-13, although there was a clear trend towards increased IL-13 responses at the one month time point for both the TMD and OMD diets (Figure 5B). The production of IL-1, IL-6, G-CSF and GM-CSF by activated PBMCs were elevated at the one month OMD and one month TMD time points, compared to the other time points (Table 1), suggesting that a change from normal to controlled diets affects these cytokine regulatory pathways.

**Figure 6:**
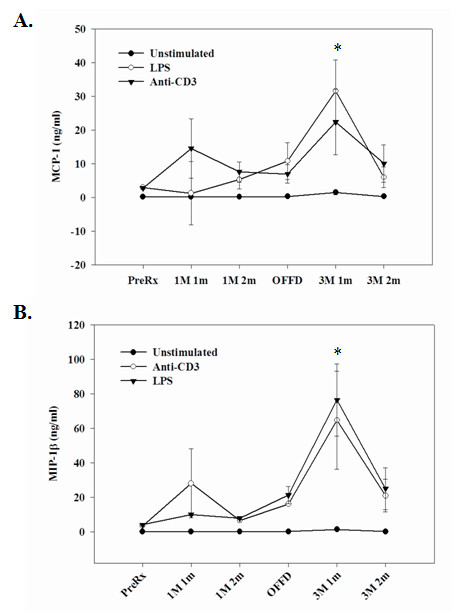
**Stimulated PBMCs derived from subjects on OMD and TMD diets were examined for MCP-1 and MIP-1β expression**. Production of MCP-1 and MIP-1β could be detected in the culture supernatant from un-stimulated PBMCs. The LPS-induced MCP-1 and MIP-1β release from PBMCs was significantly lower at one month time point in subjects fed OMD versus TMD (p < 0.05). The data are expressed in pg/ml (+/-SEM). The numbers of subjects (n) examined for each stage of the study are as follows: Pre-treatment/Baseline (n = 15), 1 meal/1 month (n = 8), 1 meal/2 month (n = 12), Off-diet (n = 12), 3 meals/1 month (n = 12) and 3 meal/2 month (n = 12).

We next measured the production of the proinflammatory chemokines; MCP-1 and MIP-1β by PBMCs treated with LPS and anti-CD3 mAb, and observed significantly lower production of these chemokines in subjects when on OMD and TMD diets compared to pretreatment and off diet time points (Figure 6A, B). The magnitude of the elevation of the latter proinflammatory cytokines was greater when subjects were on the TMD diet compared to the OMD diet.

## Discussion

Although there is ample anecdotal evidence of health benefits of fasting in the healthy adults, only recently have such dietary interventions been rigorously studied in laboratory animals and human subjects. Emerging evidence suggests that mice and rats maintained on repeated cycles of 24 hours with no food followed by 24 hours with free access to food (IF) lived up to 30% longer [37,38] compared to *ad libitum *fed controls. In addition, the IF animals displayed improved insulin sensitivity, reduced cancers and increased resistance of neurons and cardiac cells to oxidative and metabolic stress [17,39]. However, the effects of meal frequency and intermittent fasting on immune function in humans are unknown. Given the highly robust beneficial effects of IF in experimental models, we designed the present study in humans to test the hypothesis that a change from a usual TMD diet to an OMD weight maintenance diet would alter proinflammatory cytokine expression in circulating lymphocytes. Analysis of serum levels of the proinflammatory markers, CRP, ICAM-1 and soluble gp130 revealed no significant differences between the OMD and TMD diet groups. We also studied serum visfatin, a novel adipocytokine, which is synthesized mainly by visceral adipocytes and serves as an insulin mimetic and proinflammatory cytokine [31-33]. Similar to other proinflammmatory markers, we observed that OMD and TMD diets have no impact on serum visfatin levels in these subjects. Thus, meal frequency did not significantly affect levels of circulating pro-inflammatory markers, suggesting that a change in meal frequency does not alter the basal inflammatory state.

Because the subjects in our study were healthy and of normal weight, and so would not be expected to display elevated pro-inflammatory markers, we studied the impact of fasting on regulation of T-helper cytokines and chemokines from PBMCs isolated from the subjects at designated time points throughout the study. The isolated PBMCs were activated ex-vivo with LPS and TCR ligation. Compared to the baseline pretreatment values, we observed a large unexpected increase in cytokine secretion from stimulated cells one month after initiation of either the OMD or TMD diets. Although the precise mechanism responsible for this elevated cytokine release is unknown, it is quite feasible that departure from pre-study regular eating patterns and adherence to OMD and TMD controlled diets in both study groups made the PBMCs hyper-responsive to stimulation. As the study progressed into the second month of the controlled TMD and OMD diet periods, the stimulated cytokine levels in the subjects returned closer to the baseline suggesting a habituation and adaptation to the dietary intervention. A stress-based mechanism for the enhanced responsiveness of PBMC when subjects were on the TMD and OMD controlled diet would be consistent with previous studies have provided evidence that mild stress can enhance PBMC activation. For example, PBMCs isolated from subjects following exposure to psychosocial stress [40] or 5 min of vigorous exercise [41] exhibited increased activation of NF-κB, a transcription factor know to induce the production of various cytokines. In addition, chemotaxis and expression of cell adhesion molecules were increased in PBMCs isolated from subjects immediately after acute psychological stress [42].

This meal frequency study was the first human study in which daily calories were held constant between two diets that differed only in meal frequency (3 smaller meals versus one large meal) [28]. A large number of physiological variables were measured, including heart rate, body temperature and blood chemicals and many of these were unaffected by altering meal frequency. However, when on 1 meal per day, subjects did exhibit a significant reduction of fat mass and significant increases in levels of total, low-density lipoprotein, and high density lipoprotein cholesterol [28]. In addition, the morning glucose tolerance was found to be impaired when subjects were consuming 1 meal per day compared with 3 meals per day [29]. Fasting (morning) plasma glucose levels were also significantly elevated in subjects when they were consuming 1 meal per day compared with 3 meals per day and this 1-meal-per-day diet effect on glucose tolerance was rapidly reversed upon return to the 3-meals-per-day diet, indicating that the diet had no long-lasting effect on glucose metabolism. Besides these effects on glucose metabolism, there were no significant effects on the expression of a number of metabolic variables including plasma levels of ghrelin, adiponectin, resistin or BDNF. In the current study, we have further examined these subjects for alterations in inflammatory and immune parameters with changes in diet and noted that meal frequency changes do cause transient increases in TCR- and TLR4 (LPS)-mediated expression of several cytokines and that the magnitude of these alterations is less when subjects consume OMD versus TMD. Interesting patterns of expression were revealed where there appears to be more of a stress response at the initiation of the diets at the one month time interval. More pronounced cytokine expression changes were noted in the 1 month time period of the TMD subjects versus the same subjects given OMD supporting our conclusions that reduced meal frequency can have an impact on PBMC-derived cytokine expression between OMD and TMD subjects. Despite the observed changes in metabolic parameters reported in this study [28,29], we failed to note any significant correlations or associations between the observed cytokine changes in expression with diet and BMI or circulating levels of glucose, insulin, leptin, ghrelin, adiponectin, resistin or BDNF.

In the first month after the initiation of the diets, we observed a robust increase in IFN- γ and TNF-α release from PBMCs in subjects fed TMD that was significantly greater than when the subjects were on the OMD diet. In a smaller study, the immune cells derived from fed healthy subjects and stimulated ex vivo by TCR ligation produced significantly higher levels of IFN- γ with lower IL-4 levels compared to overnight fasted individuals [43] suggesting that fasting promotes Th2 responses. In our study no significant differences were observed in Th2 cytokines from OMD and TMD groups; however similar to the previous study [43], we observed lower levels of Th1 cytokines in subjects when they were on the OMD diet. We have also recently reported that alternate day calorie restriction in overweight adults with asthma results in marked decline in circulating TNF-α levels with improvement in pulmonary functions and measures of quality of life [27]. Recent studies suggest that in addition to Th1 and Th2 there is a subset of IL-17 producing T helper cells (Th17) that are involved in various autoimmune inflammatory disorders [36,44]. Compared to the TMD diet, we observed significantly reduced IL-17 secretion from stimulated T cells derived from subjects during the OMD diet. It has been suggested that IL-23 is a key regulator of IL-17 production in T cells [35,36]. We observed that PBMCs from subjects on TMD for 2 months expressed 6-fold higher levels of IL-23 mRNA compared to the baseline level, with no difference in IL-17 receptor expression. Recent studies suggest that fasting induced down-regulation of leptin protects against autoimmune encephalomyelitis [45,46]. Our data suggest for the first time that fasting can also down regulate the IL-17 pathway in human T cells and hence could modify autoimmune processes. Interestingly, compared to the TMD diet group, we also observed a significant reduction in MCP-1 and MIP-1β production from stimulated PBMCs derived from OMD fed subjects suggesting a tight coupling of metabolic and immune systems.

In conclusion, our study demonstrates that upon specific challenges ex vivo, leukocytes cells derived from subjects on an OMD diet respond with lower pro-inflammatory cytokine production. The immune compartment appears to be exquisitely sensitive to the behavioral and metabolic cues, and the application of intermittent fasting as an approach for modifying immune function to improve health warrants further study.

## Competing interests

The authors declare that they have no competing interests.

## Authors' contributions

All authors read and approved the final manuscript. VDD, HY, KSS, RSP performed the PBMC isolation, cultures, multiplex and ELISA assays and the PCR analysis in this manuscript. KSS, WVR, DJB, DLL, MPM and DDT played a role in the planning, design and performance of the clinical trial and in the evaluation of the data. VDD, MPM and DDT played a role in the writing and editing of the manuscript.
